# The Jaw-Locking Case of a Missed Tetanus Booster

**DOI:** 10.5811/cpcem.33516

**Published:** 2025-02-26

**Authors:** Erica Westlake, Katherine Billings, Ann McMoran, Katherine Selman, Sarab Sodhi

**Affiliations:** 1 Cooper University Hospital, Department of Emergency Medicine, Camden, New Jersey; 2 Inova Fairfax Hospital, Department of Emergency Medicine, Falls Church, Virgina; 3 Cooper Medical School of Rowan University, Camden, New Jersey

**Keywords:** Case Report, Tetanus, Infectious Disease, Immunization

## Abstract

**Introduction:**

Tetanus is a now rare disease due to the widespread administration of scheduled and prophylactic vaccines, making it exceptionally uncommon to appear in many emergency departments. Clinical suspicion alone is used to make the diagnosis as there are currently no immediately available diagnostic tests available to the clinician. If left unrecognized and untreated, however, tetanus can lead to airway compromise and death.

**Case Report:**

We report a case of a young male who presented to the emergency department with intermittent full body spasms and stiffness of the masseter muscles in the setting of recent assaults and lacerations weeks prior who had not received tetanus since 2008. Immediate calls were placed to infectious disease consultants and the patient was treated with intravenous immunoglobulin, tetanus immunization, metronidazole, and ceftriaxone. Further work up revealed rhabdomyolysis, elevated lactate, and unremarkable imaging.

**Conclusion:**

Following treatment, the patient’s symptoms improved to resolution with completion of therapy, effectively confirming the diagnosis of tetanus.

## INTRODUCTION

Tetanus is a serious infectious disease caused by *Clostridium tetani*. This bacterium forms a toxin which causes inhibition of gamma-aminobutyric acid neurotransmission, leading to muscle spasms resulting in airway obstruction and death. Rarely seen in countries with robust vaccination efforts, tetanus is a diagnostic challenge in the emergency department (ED). Although many patients present with open wounds, up to 30% of patients have no entry apparent.^1^ Generalized tetanus is seen in 80–84% of cases, which is characterized by full body spasms and can progress to autonomic instability.^2^ Lockjaw is the most common presenting symptom. Neonatal tetanus is much less common with current immunization guidelines for pregnancy and pediatrics. Local tetanus, making up between 2–13% of cases, is defined by muscle spasms proximal to the site of injury, and can either self-resolve over the course of weeks or progress to generalized tetanus.^2^ Cephalic tetanus, the rarest form of tetanus accounting for approximately 1–3% of cases, is localized to the face and causes flaccid paralysis rather than spasm (Table).^3^ Complications of tetanus include rhabdomyolysis, long bone fractures, respiratory compromise, and aspiration pneumonia.

## CASE REPORT

A 29-year-old male patient was brought to the ED by emergency medical services complaining of full body spasms and jaw pain. Upon initial evaluation, the patient was afebrile, and then suddenly became extremely diaphoretic, tachycardic, hypertensive and exhibited difficulty speaking. He was able to track clinicians in the room, shake his head yes or no, and was alert throughout the duration of the episode. His upper extremities and abdomen were clenched, back was arched, and neck was turned to the side. This episode resolved spontaneously, the patient was not post-ictal, and further evaluation continued. The patient denied taking any substances prior to presentation, denied medications, and denied past medical history.

Physical examination was notable for multiple wounds on the right hand, including a surgical site with suture on the dorsal fifth digit of the right hand, open wound on the right palm, and healing laceration of the left face. None of the wounds had erythematous bases or purulent discharge.

The differential for this patient included: serotonin syndrome, neuroleptic malignant syndrome, dystonic reactions, toxidrome, rabies, orofacial infection, and strychnine poisoning, all of which were less likely due to self-resolving episodes.^1^,^3^ Seizure was considered less likely due to bilateral extremity involvement and the ability of patient to track and to respond throughout episodes. Benztropine therapy was a consideration to differentiate between tetanus and a drug-induced dystonic reaction.^4^

Labs were significant for elevated creatine kinase (CK) to 2678 Units per liter (U/L) (reference range: 0–200 U/L), creatinine 1.28 milligrams per deciliter (mg/dL) (0.6–1.20 mg/dL), lactate 9.2 millimoles (mmol)/L (0.5–2.2 mmol/L), and calcium 9.6 mg/dL (8.5–10.5 mg/dL). Computed tomography head showed no acute intracranial pathology.

Chart review demonstrated recent admission two months prior for a hand infection requiring operative washout and intravenous antibiotics, and other visits for intoxication over the past year. Per chart review, the patient had received full initial tetanus series in childhood and booster in 2008, 14 years prior to this presentation.

Approximately two hours after initial presentation, the patient had another episode of spasm, for which 2.5 mg midazolam was provided to cease the spasm. Infectious disease was consulted with concern for tetanus given open wounds, intermittent body muscle spasms with stiffness of the masseter muscles, back arching, and rigid paralysis. The infectious disease team agreed with the plan of intravenous immunoglobulin, tetanus immunization, and antibiotic coverage with metronidazole and ceftriaxone. Neurology was consulted as seizure was on the differential; on evaluation the neurology team agreed that seizure was unlikely, and recommended assessment for stiff person syndrome if there was no improvement with tetanus treatments. The patient was ultimately admitted to the critical care service for close airway monitoring.

**CPC-EM Capsule**

What do we already know about this clinical entity?

*We understand the pathophysiology, causative organism, and different presentations of tetanus*.

What makes this presentation of disease reportable?

*This case is reportable due to the rare nature of tetanus. Reports such as this are often the only exposure most clinicians have to this pathology*.

What is the major learning point?

*This case discusses the immediate diagnosis and treatment of tetanus in the emergency department*.

How might this improve emergency medicine practice?

*This case calls to attention the opportunity for improved tetanus vaccination in the emergency department as well as provides a guideline for treatment of tetanus*.

After the patient’s admission, he did not have any more episodes of muscle spasms, his CK down-trended and the patient was transferred to the medical floor. He was able to be transitioned to oral antibiotics and was provided strict return precautions and instructions for completing tetanus immunization series via return to the ED or through primary care provider referral provided. Five days after hospital discharge, the patient reported to the ED with leg spasms and reported he had been unable to pick up antibiotics from the pharmacy. He was provided additional antibiotics and prescriptions. No further encounters were recorded.

## DISCUSSION

This case highlights several key points: early tetanus course can present as intermittent spasms with autonomic instability, aggressive management early can lead to favorable outcomes, and the ED has an opportunity for continued tetanus immunization, especially in vulnerable populations. There is data to suggest a decline in protective antibodies against tetanus with age, especially at age 60 and beyond. 5 Vaccination response declines with age as well, so despite having a booster within the last ten years, this population may not be entirely protected with the standard schedule. 5 This population often presents to the ED with trauma and falls, and tetanus could be overlooked or not administered.^5^

Tetanus is a rare diagnosis in developed countries due to widespread vaccinations. However, tetanus should be included on the differential, particularly in emergency departments that treat immigrant populations, people who have limited access to consistent health care, or patients who have not been fully vaccinated. Tetanus can have a spectrum of presentations, including the above description of intermittent spasms.^2^,^3^ Recognizing this disease process early can lead to initiation of treatment and more favorable patient outcomes. In this case, we were able to initiate treatment for tetanus prior to continuous spasms and airway compromise, leading to less invasive management of the patient. Metronidazole and ceftriaxone were chosen in conjunction with infectious disease consultation, for aerobic and anaerobic coverage, and with review of prior case reports. The role of antibiotics is to prevent multiplication of the bacteria, therefore limiting the toxin production and reducing mortality. We were able to provide resources for continuation of tetanus treatment and immunization.

An additional population which is at risk for tetanus includes patients involved in natural disasters. The emergency department is frequently activated for disaster scenarios, and therefore has a unique opportunity to evaluate and triage patients in the midst of a disaster. Tetanus outbreaks can occur in natural disasters due to risk for puncture and soft tissue injuries in conjunction with the spore forming nature of the causative organism, *Clostridium tetani*.^6^ Recognizing the need for immunization in addition to triage and treatment of injuries is important in the setting of natural disease, as well as maintaining a high clinical suspicion for tetanus the weeks following natural disasters in patients who had exposure.

## CONCLUSION

Despite vaccination rates and education, certain populations are still under-immunized or not fully protected from tetanus. These include patients who have not been compliant with vaccination status, have not had access to routine care for scheduled immunizations, victims of a natural disaster and the elderly, who are often under protected despite being vaccinated.^5^ The emergency department provides a unique opportunity to increase immunity for at-risk groups and recognize the spectrum of presentations of tetanus. This case demonstrates, however, a patient who had had regular encounters with the healthcare system, including for treatment of the wounds that are presumed to have been the entry source for the tetanus toxin, and yet his tetanus vaccination status had been overlooked. This illustrates the need for attention to the mundane topic of tetanus vaccine status and the role of the ED in preventative healthcare.

## Figures and Tables

**Table:** Types of Tetanus

Type	Prevalence
Generalized	80–84%
Localized	2–13%
Cephalic	1–3%
